# Patterns of sequence polymorphism in the *fleshless berry *locus in cultivated and wild *Vitis vinifera *accessions

**DOI:** 10.1186/1471-2229-10-284

**Published:** 2010-12-22

**Authors:** Cléa Houel, Rémi Bounon, Jamila Chaïb, Cécile Guichard, Jean-Pierre Péros, Roberto Bacilieri, Alexis Dereeper, Aurélie Canaguier, Thierry Lacombe, Amidou N'Diaye, Marie-Christine Le Paslier, Marie-Stéphanie Vernerey, Olivier Coriton, Dominique Brunel, Patrice This, Laurent Torregrosa, Anne-Françoise Adam-Blondon

**Affiliations:** 1 Unité mixte de Recherche en Génomique Végétale (URGV), INRA UEVE ERL CNRS, 2 rue Gaston Crémieux, 91 057 Evry cedex, France; 2 Unité INRA Etude du Polymorphisme des Végétaux (EPGV), 2 rue Gaston Crémieux, 91 057 Evry cedex, France; 3 CSIRO Plant Industry, PO BOX 350, Glen Osmond SA 5064, Australia; 4 Unité mixte de Recherche Diversité et Adaptation des Plantes Cultivées (DiaPC), INRA SupAgro, 2 place Pierre Viala, 34 060 Montpellier Cedex, France; 5 Unité mixte de Recherche Amélioration des Plantes et Biotechnologies Végétales (APBV), INRA Agrocampus Rennes, Plate-forme cytologique moléculaire, 35 653 Le Rheu Cedex, France; 6 Unité mixte de Recherche Biologie et Génétique des Interactions Plantes-Agents Pathogènes (BGPI), INRA SupAgro CIRAD, 2 place Pierre Viala, 34 060 Montpellier Cedex, France

## Abstract

**Background:**

Unlike in tomato, little is known about the genetic and molecular control of fleshy fruit development of perennial fruit trees like grapevine (*Vitis vinifera *L.). Here we present the study of the sequence polymorphism in a 1 Mb grapevine genome region at the top of chromosome 18 carrying the *fleshless berry *mutation (*flb*) in order, first to identify SNP markers closely linked to the gene and second to search for possible signatures of domestication.

**Results:**

In total, 62 regions (17 SSR, 3 SNP, 1 CAPS and 41 re-sequenced gene fragments) were scanned for polymorphism along a 3.4 Mb interval (85,127-3,506,060 bp) at the top of the chromosome 18, in both *V. vinifera cv*. Chardonnay and a genotype carrying the *flb *mutation, *V. vinifera cv*. Ugni Blanc mutant. A nearly complete homozygosity in Ugni Blanc (wild and mutant forms) and an expected high level of heterozygosity in Chardonnay were revealed. Experiments using qPCR and BAC FISH confirmed the observed homozygosity. Under the assumption that *flb *could be one of the genes involved into the domestication syndrome of grapevine, we sequenced 69 gene fragments, spread over the *flb *region, representing 48,874 bp in a highly diverse set of cultivated and wild *V. vinifera *genotypes, to identify possible signatures of domestication in the cultivated *V. vinifera *compartment. We identified eight gene fragments presenting a significant deviation from neutrality of the Tajima's D parameter in the cultivated pool. One of these also showed higher nucleotide diversity in the wild compartments than in the cultivated compartments. In addition, SNPs significantly associated to berry weight variation were identified in the *flb *region.

**Conclusions:**

We observed the occurrence of a large homozygous region in a non-repetitive region of the grapevine otherwise highly-heterozygous genome and propose a hypothesis for its formation. We demonstrated the feasibility to apply BAC FISH on the very small grapevine chromosomes and provided a specific probe for the identification of chromosome 18 on a cytogenetic map. We evidenced genes showing putative signatures of selection and SNPs significantly associated with berry weight variation in the *flb *region. In addition, we provided to the community 554 SNPs at the top of chromosome 18 for the development of a genotyping chip for future fine mapping of the *flb *gene in a F2 population when available.

## Background

Berry size is an important trait in relation to both yield (table grapes) and quality (wine grapes). Indeed, the flavor in wine results from the ratio of skin to flesh, the former being the source of most aromatic and tannins compounds, the second providing the organic acids and the sugars [1].

The genetic and molecular basis of fleshy fruit size variation have been studied in depth in tomato during the last two decades, using a large panel of diverse resources that made tomato a model species for fleshy fruit crops [2,3]. Introgression lines between wild and cultivated genotypes [4-6], near isogenic lines (NILs) [7] and artificial or natural mutants [2,8] have been created and used to study the genetic basis of fruit size variation showing that a large part of it is controlled by less than ten loci. The physiological mechanisms involved have been related to the control of (i) the cell number in the pericarp, as for the *fw2.2 *locus [9,2,10], (ii) the locule number [2,11], (iii) the late endo-reduplication in pericarp cells [12,10] and (iv) the cell wall plasticity in relation to the cell expansion [10]. All these advances in tomato are useful to assist the study of similar trait in other crops with fleshy fruits, less amenable to genetic studies, such as perennial fruit trees. Indeed, encouraging results have already shown syntheny within Solanaceae species for Quantitative Trait Loci (QTL) controlling fruit size [2]. However, the degree of transferability of knowledge from tomato to non-Solanaceae species remains an open question.

Like tomato, grapevine (*Vitis vinifera*) produces fleshy fruits and a large difference in fruit size between wild and cultivated genotypes can be observed [13]. Indeed the wild *V. vinifera *genotypes produce mature berries weighting less than 1g while berries of some table grape varieties can weigh 10 g and more [14]. The growth of a grapevine berry roughly follows the same pattern as for tomato fruit: the first phase of fruit development is due to both cell multiplication and cell expansion, followed by a lag phase corresponding to a major cell metabolic shift and a second phase of fruit growth, mostly explained by cell expansion but without evidence of endoreduplication [15]. The genetic analysis of grape berry size variation is more difficult than in tomato, due to the long biological cycle of the plant, to the high level of heterozygosity of the genome and to the large field area usually required for plant growth, which makes experiments in controlled environment more costly [16-19]. In addition, berry size studies have often been performed on population segregating for seedlessness, with a strong negative correlation between the two traits: the seedless berries are in average smaller than the seeded berries [16-19]. Up to now, it has not been possible to establish the relationship between QTL for berry size and processes like cell multiplication or cell enlargement.

A natural mutant of *V. vinifera cv*. Ugni Blanc, which produces fleshless berries similar to those observed in wild genotypes, was identified as an opportunity to get insights into the control of berry development and berry size [20]. It has been shown that the drastic phenotypic changes observed in berry development are controlled by a dominant mutation in the *fleshless berry (flb*) gene [21]. Like the *fw2-2 *gene in tomato, the *flb *gene impairs cell divisions in the developing ovaries [21]. The closest genetic marker linked to the *flb *mutation defines a 6 cM region located at the top the chromosome 18 that corresponds to a physical distance of 948 kb according to the last version of the grapevine genome assembly http://urgi.versailles.inra.fr/index.php/urgi/Species/Vitis/Resources; in this region, no homolog to the *fw2-2* gene has been identified. Considering the importance of berry size for wine quality, a fine mapping of the *flb *mutation was thus started for its molecular identification.

Here we describe our efforts in reducing the genome interval of the region carrying the *flb *mutation. We first started by a classical genetic mapping approach. We showed that the mutation is located on a completely homozygous portion of chromosome 18 in Ugni Blanc mutant. No marker could thus be found in coupling with the mutation and the classical approach was abandoned.

We therefore started another approach similar to the one previously proposed for *fw2-2 *gene in tomato [9]. Since the berries of Ugni Blanc mutant mimic wild *V. vinifera *berries (both types of berries have little to no flesh and carry round shaped seeds typical of wild genotypes) [13,20,22], we hypothesized that *flb *gene could have been one of the genes selected during the domestication process of grapevine. If so, a signature of selection or selective sweep could be found around this gene. Under this assumption, we performed a preliminary scan of the sequence polymorphism of the *flb *region in a collection cultivated and wild grapevine genotypes.

## Methods

### Plant material

The genotypes used in the present study were collected in the French National Grapevine Germplasm Collection (Domain of Vassal, Montpellier, France; http://www1.montpellier.inra.fr/vassal/) and are listed in additional file 1. Twenty-six of them were chosen to maximize the genetic diversity of the cultivated *Vitis vinifera *compartment [23]. Seven other genotypes belonging to the wild *Vitis vinifera *compartment were chosen because they had well characterized wild-type phenotypes as well as wild-type diverse SSR profiles and because they originated from different countries (8500Mtp3 from Tunisia, 8500Mtp9 and 8500Mtp38 from Germany and the rest from France; [additional file 1]. Five genotypes were added to the sample: the inbred line INRA Colmar lignée PN40024 (PN40024; reference genome; maintained at INRA Colmar, France), Chardonnay, Ugni Blanc, Ugni Blanc mutant and Pinot Noir clone ENTAV-INRA777 (PN777; maintained at the French Institute for Grapevine and Wine; Domaine de l'Espiguette, Le Grau du Roi, France). The average berry weight at maturity was measured from 30 berries cut at the pedicel base 40 days after *véraison *[additional file 1].

### DNA extraction

Total genomic DNA was extracted from 1 g of young leaves according to the DNeasy Plant Maxi Kit (Qiagen) with the following modifications: 1% polyvinylpyrrolidone (PVP 40 000) and 1% (v/v) βmercaptoethanol were added to buffer AP1. The clarified lysate recovered after filtration with the QIA-shredder Maxi spin column (step 12) was extracted with one volume of phenol:chloroform:isoamyl alcohol (25:24:1) and then with one volume of chloroform:isoamyl alcohol (24:1). From this step forward, the supernatant was treated following the Qiagen instructions.

### Gene fragments amplification and sequencing

Based on the genome annotation provided by Jaillon et al [24], 86 primer pairs were designed using the Primer 3 software v.0.4.0 [25] in order to amplify every 13 kb in the *flb *region, a gene fragment of approximately 1300 bp [additional file 2]. In order to estimate the nucleotide diversity at the whole genome scale, seventy-seven other primer pairs were designed on genes chosen randomly along the genome, taking care that each chromosome was represented by three to five fragments [additional file 3]. The amplicon sequences were then aligned on the last 12× version of the genome sequence http://urgi.versailles.inra.fr/cgi-bin/gbrowse/vitis_12x_pub/ and some of them did not correspond to a gene model anymore. Settings for Primer 3 were: optimum Tm = 55°C, minimum Tm = 53°C, maximum Tm = 57°C, max 5' self complementarity = 4, max 3' self complementarity = 1. In order to amplify all the genotypes while at the same time detecting a maximum of polymorphism, all the primers were designed in exons at both sides of introns. Universal primers T7/SP6 extensions were added to the primers to allow sequencing. All PCR amplifications were carried out as described by Philippe et al [26].

### Microsatellite, CAPS and SNP genotyping

The markers genotyped are listed and described in additional file 2. Cleaved Amplified Polymorphic Sequence (CAPS) genotyping was performed as described by Salmaso et al [27]. The Australian Genome Research Facility (AGRF) carried out Simple Sequence Repeats (SSR) and Single Nucleotide Polymorphism (SNP) analysis. SNP were scored using the MassARRAY^® ^iPLEX Gold assay with MALDI-TOF MS detection (Sequenom) and SSR analysis was performed as previously described by Thomas et al [28].

### Quantitative PCR assay

Two primer pairs were designed to amplify genomic DNA. The first pair (TCTGATGCGATGTTAGTGGT and TCTGGTATTGGCGTTGG) targeted a unique gene (*FL*) in the *flb *region (gene ID GSVIVG01013466001). The second pair (AACTGGATTGAAGGGCGTGG and AGGTTCTTGAGCATGTTAAGC) targeted the *3-hydroxy-3-methylglutaryl-coenzyme A reductase *(*HMGCoA*) gene family, which members are respectively located on the chromosomes 4, 3 and 18 (gene id GSVIVG01026444001, GSVIVG011023852001, GSVIVG01013435001). Real-time PCR conditions were conducted as described by Reid et al [29], with half quantity of PCR mix and of DNA. The PCR efficiencies were determined for each gene and were 92.3% and 97.2% for *FL *and *HMGCoA *respectively. In order to compare the initial DNA quantity between genotypes in the *flb *region, the DNA quantity based on *FL *gene data was normalised using the DNA quantity of *HMGCoA *genes as a reference.

### BAC-FISH assay

Roots tips of 0.5-1.5 cm length were treated in the dark with 0.04% 8-hydroxiquinoline for 2 h at 4°C followed by 2 h at room temperature to accumulate metaphases. They were then fixed in 3:1 ethanol-glacial:acetic acid for 12 hours at 4°C and stored in ethanol 70% at -20°C. They were washed in 0.01 M citric acid-sodium citrate pH 4.5 buffer for 15 min and then digested in a solution of 5% Onozuka R-10 cellulase (Sigma), 1% Y23 pectolyase (Sigma) at 37°C for 1 h. Digested root tips were then carefully washed with distilled water for 2 h. One root tip was transferred to a slide and macerated in a drop of 3:1 fixation solution (ethanol-glacial:acetic acid). Chromosome spreads were prepared for hybridization as described by

Leflon et al [30]. VV40024H140P14 Bacterial Artificial Chromosome (BAC) clone (available at http://cnrgv.toulouse.inra.fr) was labelled by random priming with biotin-14-dUTP (Invitrogen). The ribosomal probe used, as a control of hybridation, was pTa-71 which contains a 9 kb *Eco*RI fragment of ribosomal DNA repeat unit (rDNA 18S-5.8S-26 S genes and spacers) isolated from *Triticum aestivum *[31]. The probe pTa-71 was labelled with Alexa-488 dUTP (Invitrogen) by random priming. Fluorescence *In Situ *Hybridization (FISH) experiments and capture of fluorescence images were done as described by Leflon et al [30].

### Sequence data analysis, estimation of parameters of diversity and linkage disequilibrium

Raw data were aligned and trimmed using either the Genalys v.2.8.3b software for Macintosh [32] or the Staden software v.2.0.0 [33]. They were manually edited and INsertions/DELetions (INDELs) were added when needed. Single Nucleotide polymorphisms (SNPs) were detected, confirmed, and imported into the SNiPlay database http://sniplay.cirad.fr. Nucleotide diversity (π), number of segregating sites (θ), number of haplotype (H), haplotype diversity (Hd), and Tajima's D test of neutral evolution [34] were obtained for each gene fragment using the DnaSp V5.10 software http://www.ub.edu/dnasp/. Eventually, the total value of each parameter was calculated as a weighted average for the whole data set. As all the gene fragments along the *flb *region were separated in average by 12 kb (from 3 to 57 kb), it was not possible to reconstitute the haplotypes for the entire *flb *region in order to estimate the Linkage Disequilibrium (LD). Roger and Huff [35] showed that the genotypic correlation coefficient (based on genotypic data) is a good estimator of the haplotypic correlation coefficient. LD was therefore estimated over the entire studied region as the square of the genotypic Pearson correlation coefficient (r^2^) together with its p-value using a homemade R program. The results were visualised using in homemade Perl scripts.

### Association genetics

A structured association test was carried out using TASSEL software http://www.maizegenetics.net/bioinformatics. The population structure was calculated using STRUCTURE software [36] using the genotypes at 20 SSR markers well spread along the 19 chromosomes (Le Cunff et al, 2008; R. Bacilieri unpublished results; [additional file 1]). A General Linear Model test, which takes into account the structure of the sample, was performed between the SNP markers in the *flb *region with a allelic frequency >0.05 and the average berry weight at maturity. A Bonferroni correction was applied to control false-positives: a SNP marker was declared significant if its Bonferroni p-value was less than 0.05.

## Results

### A 1 Mb region at the top of chromosome 18 is homozygous in Ugni Blanc and the fleshless berry mutant

**Figure 1:**
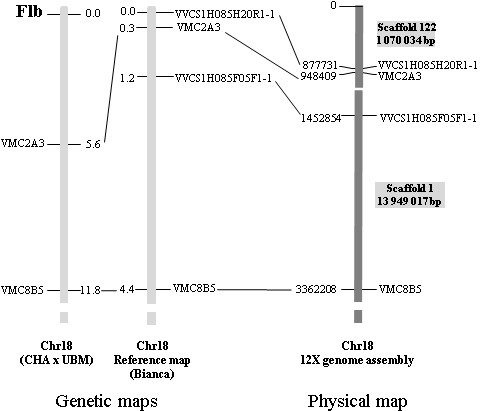
**Localization of the region containing the *flb *locus on the grapevine reference genome sequence**. On the left, the map published by Fernandez et al [21] (CHA: Chardonnay, UBM: Ugni Blanc mutant) aligned to one of the informative parental maps used for the genome assembly (A. Canaguier, unpublished results). On the right, alignment to the 12× genome sequence of the top of chromosome 18 http://urgi.versailles.inra.fr/index.php/urgi/Species/Vitis/Resources. The coordinates in kb correspond to the start of the marker sequence on the chromosome sequence. The scaffolds that constitute this part of the chromosome 18 are drawn.

The *flb *mutation was localised by Fernandez et al [21] at the top of chromosome 18, above the markers VMC2A3 and VMC8B5 on the consensus map of a progeny of Chardonnay by Ugni Blanc mutant. However, the *flb *locus was mapped indirectly relative to VMC2A3 that segregated in Chardonnay and not in Ugni Blanc mutant. For the purpose of finding polymorphic markers in Ugni Blanc mutant above VMC2A3, we aligned the genetic map to the grapevine reference genome sequence [24] in order to identify SSR and SNP markers segregating in the Ugni Blanc mutant. This region corresponded to 948 kb on chromosome 18 (upper part of scaffold 122; Figure 1) where 100 predicted genes were proposed by the automatic annotation.

First, 17 SSR, three SNP and one CAPS markers were either developed or retrieved from published genetic maps [37-42] along scaffold 122 and the beginning of scaffold 1, above and below VMC2A3 [additional file 2]. All primer pairs successfully amplified Chardonnay and Ugni Blanc mutant genomic DNAs. One of them (VVS55), not targeting a single locus, was discarded. Chardonnay was heterozygous for ten of the 20 remaining markers, while Ugni Blanc mutant was always homozygous except for VVCS1H085F05F1-1, which is located after VMC2A3 (Table 1).

**Table 1:** Marker polymorphism observed between cultivars Chardonnay and Ugni Blanc mutant on the top of the chromosome 18 (12× genome assembly).

Position on the chromosome 18 (bp)						
Scaffold	Start	End	Marker name	Marker type	Chardonnay^$^	Ugni Blanc mutant^$^
122	212555	212699	VVS50	SSR	H	h
122	213864	214083	VVS51	SSR	H	h
122	226346	226575	VVS52	SSR	h	h
122	230668	230769	VVS53	SSR	H	h
122	308176	308256	1036L11F	SNP	h	h
122	321067	321135	VMC3E5	SSR	h	h
122	388123	388423	VVIN03	SSR	h	h
122	423185	423271	1038A12F	SNP	h	h
122	494374	494464	VVS54	SSR	H	h
122	497723	498045	IN0954	CAPS	h	h
122	670015	670200	VVS56	SSR	h	h
122	804498	804634	VVS57	SSR	H	h
122	877751	878077	VVCS1H085H20R1-1	SSR	h	h
122	895761	895846	1073P15R	SNP	h	h
122	901775	901934	VVS58	SSR	H	h
122	948267	948387	VMC2A3*	SSR	H	h
1	1226489	1226647	VVCS1H066N21R1-1	SSR	H	h
1	1297892	1298020	C011	SSR	H	h
1	1452854	1453153	VVCS1H085F05F1-1	SSR	H	H
1	2912753	2913088	VVIB31	SSR	h	h
1	3505999	3506060	VVIV16	SSR	H	h

^$ ^H: for heterozygous marker and h: for homozygous marker

* From Fernandez et al [21]

In order to find new heterozygous markers in the *flb *region, we decided to carry out a re-sequencing approach. Thirty primer pairs were designed along this region [additional file 2], 24 above the SSR marker VMC2A3 and six below. Twenty out of 24 primer pairs (above VMC2A3) successfully amplified the PN40024 genomic DNA and were thus used to sequence the corresponding gene fragments in Chardonnay, Ugni Blanc and Ugni Blanc mutant. We decided to sequence also Ugni Blanc in order to check if the homozygosity of the *flb *region was specific to the mutant or already present in the wild type.

The 26 fragments of 1300 bp in average were sequenced either only in forward or also in reverse direction, leading to 41 sequences of 161 to 1700 bp long (Table 2), heterozygous INDELs or short repeats leading to the shorter sequences. In total, we analyzed 23,562 bp in Chardonnay and 29,638 bp in Ugni Blanc and Ugni Blanc mutant. This difference was the first observed contrast between Chardonnay and Ugni Blanc, due to a different level of heterozygosity. Comparing the sequences of Chardonnay and Ugni Blanc, 74 polymorphisms were identified (63 SNPs and 11 INDELs). Out of these, 10 differences correspond to homozygous SNPs or INDEL in both samples, while 64 differences correspond to SNPs heterozygous in Chardonnay and homozygous in Ugni Blanc. No heterozygous SNPs or INDELs were observed in Ugni Blanc and its mutant; we deduced that the homozygosity of this region derived from Ugni Blanc. Only Ugni Blanc mutant sequences were considered in the subsequent experiments.

**Table 2:** Sequence polymorphism observed between cultivars Chardonnay and Ugni Blanc mutant on the top of the chromosome 18 (12× genome assembly).

Position on the 12× genome assembly (bp)			Fragment name	Number of extremities sequenced	Sequence Length		Homozygous polymorphic sites between Chardonnay and Ugni Blanc mutant		Chardonnay: number of heterozygous		Ugni Blanc mutant: number of heterozygous	
								
Scaffold	Start	End			Chardonnay	Ugni Blanc mutant*	SNP	INDEL	SNP	INDEL	SNP	INDEL
122	85127	85871	VVC2982A	2	1,553	1,553	0	0	2	0	0	0
122	161551	161929	VV05806A	2	1,168	1,168	0	0	0	0	0	0
122	211001	211674	VVC2974A	1	969	969	0	0	4	0	0	0
122	261445	262084	VV05805A	2	1,562	1,562	0	0	10	0	0	0
122	299201	299664	VVC2967B	2	911	911	0	0	2	0	0	0
122	321452	321822	VV05803A	2	1,077	1,077	0	0	7	0	0	0
122	372496	372799	VV05800A	2	683	683	1	0	2	0	0	0
122	382744	382940	VVC2956A	2	452	876	0	0	2	1	0	0
122	399382	399793	VVC2953A	2	769	1,505	0	0	0	1	0	0
122	429725	431077	VV05799A	2	1,539	1,539	2	0	1	0	0	0
122	497378	497760	VVC2942A	2	933	1,255	0	0	5	1	0	0
122	510613	510723	VV05798A	1	1,464	1,464	0	0	4	0	0	0
122	549494	550104	VV05796A	2	909	909	1	0	2	0	0	0
122	615081	615296	VV05793A	1	407	1,057	0	0	1	1	0	0
122	668381	668534	VV05788A	1	914	1,053	0	0	2	1	0	0
122	702907	703637	VV05785A	1	1,090	1,446	0	0	0	0	0	0
122	776756	777088	VV05782A	1	413	1,434	2	0	2	1	0	0
122	818661	819292	VV05781A	1	830	1,495	0	0	1	1	0	0
122	898379	898848	VV05779A	1	1,565	1,565	1	0	1	0	0	0
122	928463	929045	VV05777A	2	1,030	1,520	1	0	1	1	0	0
**122**	**949921**	**950653**	**VV05775A**	**1**	**755**	**1,116**	**1**	**0**	**1**	**0**	**0**	**0**
**122**	**1009539**	**1010696**	**VVC2869A**	**1**	**137**	**498**	**0**	**1**	**0**	**1**	**0**	**0**
**122**	**1054896**	**1056021**	**VVC2865A**	**1**	**136**	**136**	**0**	**0**	**1**	**1**	**0**	**0**
**1**	**1084916**	**1085337**	**VVC15574A**	**2**	**161**	**161**	**0**	**0**	**3**	**0**	**0**	**0**
**1**	**1098027**	**1099246**	**VVC15572A**	**2**	**1,700**	**1,700**	**0**	**0**	**0**	**0**	**0**	**0**
**1**	**1104978**	**1106307**	**VVC15571A**	**2**	**435**	**986**	**0**	**0**	**0**	**0**	**0**	**0**

				41	23,562	29,638	9	1	54	10	0	0

* The column Ugni Blanc mutant stands for both Ugni Blanc and Ugni Blanc mutant, as no differences were observed between them.

The table lines in bold characters correspond to the sequence fragments below marker VMC2A3.

In total, 62 regions (17 SSR, three SNP, one CAPS and 41 re-sequenced gene fragments) were scanned for polymorphism both in Chardonnay and Ugni Blanc mutant along a 3.4 Mb interval (85,127-3,506,060 bp) in the *flb *region (scaffold 122 and the beginning of scaffold 1). This allowed showing a nearly complete homozygosity in Ugni Blanc mutant and as expected, a high level of heterozygosity in Chardonnay.

**Figure 2:**
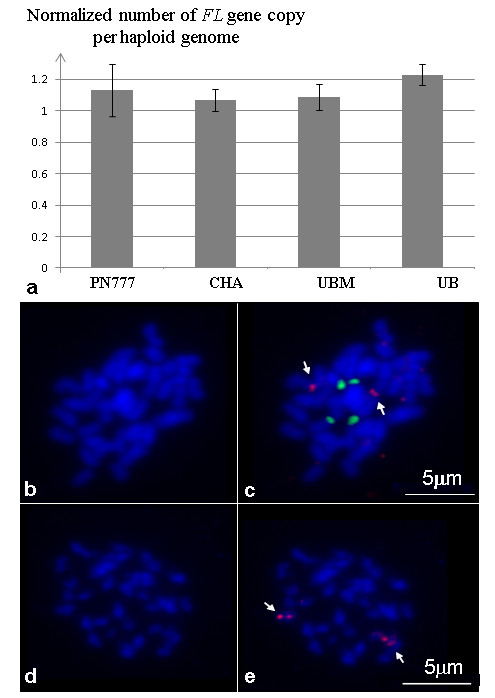
**Experimental demonstration of homozygosity of the *flb *region in Ugni Blanc mutant**. **(a) **Estimation of the number of *FL *gene copy after normalization in Pinot Noir (PN777), Chardonnay (CHA) Ugni Blanc (UB) and Ugni Blanc mutant (UBM). **(b-c) **Double fluorescence in situ hybridization (FISH) with BAC clone VV40024H140P14 (red) and pTa-71 (green) as a control, on mitotic metaphase chromosomes of Ugni Blanc mutant and **(d-e) **FISH signals of BAC clone VV40024H140P14 (red) on mitotic chromosomes of Pinot Noir (PN777) are indicated with arrows. Chromosomes were counterstained with DAPI (blue).

To discriminate between a complete homozygosity of Ugni Blanc mutant and a large deletion of the *flb *region, two experiments were realized. First, a quantitative PCR (qPCR) assay was performed on genomic DNA from Ugni Blanc, Ugni Blanc mutant, Chardonnay, PN777 and PN40024 as controls. No difference in the estimation of the initial DNA quantity was observed when amplifying with primer pair *FL*, which targeted a gene in the *flb *region and the other primer pair *HMGCoA*, which targeted three loci elsewhere in the genome (Figure 2a; [additional file 4]). This indicated that this region is homozygous and not deleted in Ugni Blanc or Ugni Blanc mutant. The second experiment consisted in a FISH experiment with a BAC clone (VV40024H140P14) localized specifically in the *flb *region using mitotic metaphase chromosomes of Ugni Blanc mutant and PN777 as control. Chromosomes were counter stained with DAPI (Figure 2b-e) and FISH signals corresponding to VV40024H140P14 were detected on two homologous chromosomes in both PN777 and Ugni Blanc mutant (Figure 2c and 2e respectively), which confirmed that the *flb *region was not deleted in Ugni Blanc mutant.

### *Flb *region showed possible signatures of selection in the cultivated *V. vinifera *compartment

A fragment every ten to 20 kb, in the 948 kb region above marker VMC2A3 was re-sequenced in a highly diverse set of cultivated *V. vinifera *genotypes [additional file 1], in order to evidence possible traces of selection in the cultivated pool of grapevines.

Sixty-three additional primer pairs were developed; two of them being discarded because they did not amplify in PN40024 [additional file 2]. Eighty-two primer pairs (20 targeting fragments before VMC2A3, one targeting a fragment after VMC2A3 described in the former paragraph and 61 newly developed) were thus used to sequence the corresponding gene fragments in 26 cultivated *V. vinifera *and the PN40024 as control [additional file 1]. Each fragment was compared to the 12× version of the genome reference sequence, which allowed us to discard the results obtained for eight and three fragments that appeared to be either part of a false duplication in the 8× version of the genome sequence, or to the same gene in the 12× gene annotation, respectively [additional file 2]. The remaining data, from 69 sequenced regions, consisted in a total of 34,355 kb, 61% (21,161 kb) being located in predicted introns or UnTranslated Region (UTR) and 39% (13,194 kb) in exons [additional file 5]. In parallel, 77 random gene fragments spread all over the genome were chosen in order to estimate the nucleotide diversity over the whole genome, and as control for the effect of selection. These gene fragments represented 48,874 kb of total sequence, 55% (27,018 kb) located in predicted introns or UTR and 45% (21,856 kb) in predicted exons [additional file 3].

**Figure 3:**
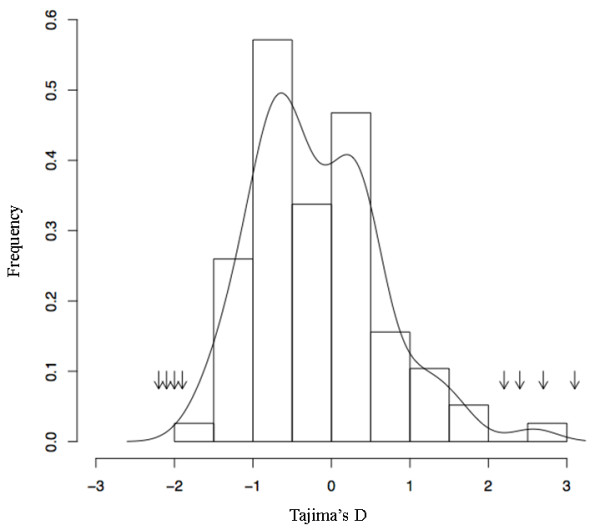
**Distribution in cultivated grapevines of the Tajima's D value calculated from the 77 genes randomly distributed across the genome**. The arrows correspond to the Tajima's D value from the eight gene fragments in the *flb *region with a significant deviation of the Tajima's D value from neutrality.

The Tajima's D parameter, was calculated for the 77 random genes and for the 69 genes from the *flb *region [additional file 3 and 5]. Eight of 69 sequenced fragments in the *flb *region showed putative traces of selection evidenced by a Tajima's D parameter significantly deviating from neutrality (Table 3). Moreover, for these fragments, the value of Tajima's D parameter was quite divergent from the average calculated for the 77 random genes (-0.1853+/-0.8117; [additional file 3]) and were found in the tails of the distribution of Tajima's D value across the genome (for α = 0.05; Figure 3). A significant negative Tajima's D value, possibly indicative of a purifying selection was observed for four out of the eight gene fragments whereas a significant positive Tajima's D value, possibly indicative of a diversifying selection, was found for the other four (Table 3).

**Table 3:** Nucleotide diversity in the wild and cultivated *Vitis vinifera* genotypes for the gene fragments along the *flb* region presenting a significant deviation from neutrality of the Tajima's D parameter.

		*Wild*		*Domesticated*			*Wine*			*Table*			
Fragment	Start 12×	π	π standard error	π	π standard error	**Tajima's D**^§^	π	π standard error	**Tajima's D**^§^	π	π standard error	**Tajima's D**^§^	
**VVC2981A**	94259	0.0007	0.0001	0.0014	0.0005	-2.0*	0.0013	0.0003	-0.5	0.0016	0.0008	-2.2**	
**VVC2946A**	444180	0.0030	0.0004	0.0043	0.0011	-1.3	0.0035	0.0017	-2.2**	0.0047	0.0016	-1.2	
**VV05791A**	638081	0	0	0.0040	0.0002	0.6	0.0034	0.0005	1.1	0.0037	0.0003	2.4*	
**VVC2897A**	682572	0.0173	0.0062	0.0044	0.0017	-2.1*	0.0070	0.0042	-2.1	0.0027	0.0004	-0.7	
**VV05785A**	702907	0.0008	0.0004	0.0003	0.0002	-1.9*	0.0003	0.0002	-1.5	0.0002	0.0001	-1.5	
**VVC2901A**	742593	0.0082	0.0035	0.0156	0.0005	3.1**	0.0157	0.0011	2.4*	0.0144	0.0079	2.6**	
**VVC2885A**	746740	0.0115	0.0037	0.0159	0.0007	2.7**	0.0167	0.0012	2.8**	0.0145	0.0020	1.5	
**VVC2892A**	808955	0.0009	0.0003	0.0109	0.0007	2.2 *	0.0102	0.0011	2.0	0.0119	0.0009	2.0	

^§ ^* 0.01<P-value < 0.05 and ** 0.001<P-value < 0.01

### Analysis of the nucleotide diversity along the *flb *region in a set of cultivated and wild *Vitis vinifera *genotypes

**Figure 4:**
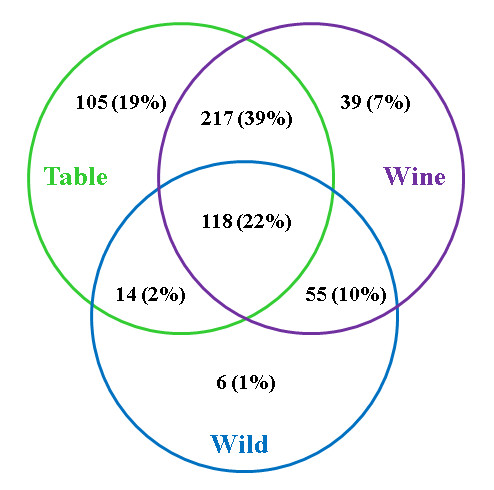
**SNP from the *flb *region in wild and cultivated grapevines**. Venn diagram showing the distribution of the 554 non-redundant SNPs found in the 948 kb region at the top of chromosome 18 in the sets of wild and domesticated table and wine *V. vinifera *genotypes.

**Figure 5:**
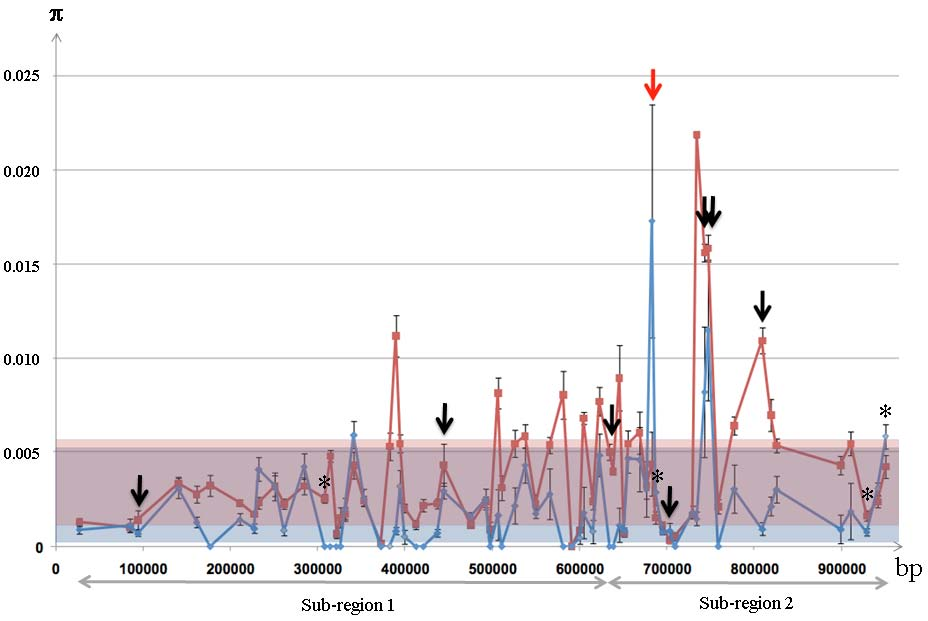
**Nucleotide diversity in wild and cultivated grapes along the *flb *region**. Nucleotide diversity (π) in wild (blue line) and cultivated grapes (red line) along the *flb *region. The standard deviation of the π parameter in the whole genome is represented by a blue and red box for wild and cultivated genotypes respectively. Genes under selection in the cultivated pool of genotypes are indicated with black arrows and the gene under purifying selection showing higer diversity in wild genotypes than in cultivated genotypes with red arrows. The two sub-regions with regard to LD patterns are underlined with grey arrows. Gene fragments with SNP significantly associated with berry weight variation are highlighted with a star.

The 69 gene fragments from the *flb *region and the 77 random gene fragments spread all over the genome were sequenced in seven diverse wild *Vitis vinifera *genotypes, in order to compare the nucleotide diversity in the cultivated and wild pools of genotypes. The diversity parameters calculated for each fragment in the two different subsets of individuals, are presented in additional files 3 and 5 and summarized in Table 4. All the indicators of genetic diversity (number of segregating sites, number of haplotypes, and nucleotide diversity: π) were higher in average (roughly doubled pi = 0.0020 *vs *0.0041; [additional file 5]) in the whole sample of domesticated genotypes in comparison to the sample of wild genotypes in the *flb *region. This hold true when each of the wine and table grape sub-compartments of cultivated grapes were compared with the wild compartment, with less unbalanced numbers of individuals in each pairwise comparison (Table 3). Compared to a similar number of re-sequenced fragments spread all over the genome, there was a slightly lower diversity among the wild genotypes in the *flb *region than in the rest of the genome, which was not the case in the cultivated compartment (Table 4). Moreover, we observed very few specific segregating sites between the wild and the cultivated compartment in the *flb *region (out of 554 SNP sites, only six were specific to the wild compartment; Figure 4; [additional file 5]). Nucleotide diversity varied along the *flb *region, also depending on the pool of genotypes considered (Figure 5; [additional file 5; additional file 6] and was locally higher in the cultivated compartment than in the wild compartment (Figure 5). This probably reflected the fact that 18 out of 69 fragments showed no sequence polymorphism among the wild genotypes [additional file 5], whereas only one fragment was monomorphic in the domesticated compartment (VV05795A). This was not the case for the 77 random fragments [additional file 3]. In addition, we found that the wine cultivar Orbois, like Ugni Blanc, was completely homozygous specifically in the *flb *region (data not shown).

**Table 4:** Summary of the sequence polymorphism observed in cultivated and wild *V. vinifera* genotypes for 69 sequence fragments along 948 kb in the *flb* region and for 77 sequence fragments spread along the whole genome.

	Average number of genotypes/fragment	Average number of segregating sites/fragment	Average number of haplotypes/fragment	Average and standard deviation of π	
***Wild (n = 7)***					
*Flb *region	5.9	2.8	2.2	0.0020 +/- 0.0006	
Whole genome	6.7	4.8	3.6	0.0027 +/- 0.0025	

***Cultivated (n = 26)***					
*Flb *region/Wine (n = 15)	10.5	6.2	4.3	0.0035 +/- 0.0007	
*Flb *region/Table (n = 11)	12.5	6.6	4.7	0.0035 +/- 0.0007	
*Flb *region/Wine + Table	24.8	8	5.8	0.0041 +/- 0.0004	
Whole genome/Wine + Table	27.0	10.1	8.3	0.0035 +/- 0.0023	

Under the hypothesis that *flb *was one of the genes under selection during grape domestication, we expected to find traces of selection in the cultivated compartment associated with a difference of nucleotide diversity between the cultivated and wild compartments. Eight sequenced gene fragments in the *flb *region were particularly interesting because they showed such possible traces of selection in the cultivated pool of genotypes (previous paragraph; Table 3). Four out of the eight gene fragments showed differences in nucleotide diversity between the two compartments (VV05791A, VVC2897A, VVC2901A and VVC2901A; Table 3). However, the wild *Vitis vinifera *sample showing over all the genome a lower diversity than the cultivated *Vitis vinifera *sample, we could conclude to a significant nucleotide diversity difference between wild and cultivated compartment only in the case where there was a decreasing of nucleotide diversity in the cultivated sample in comparison to the wild sample. Only one out of eight gene fragments (VVC2897A) showed such significant higher nucleotide diversity (π) in the wild compartment compared to the cultivated compartment. This gene encodes a putative glyceraldehyde-3-phospho-dehydrogenase (Table 3). VVC2897A was re-sequenced in Ugni Blanc and Ugni Blanc mutant, showing no polymorphism in the part of the coding region they contained (data not shown).

### *Flb *region showed significant LD and a possible association with berry size variation

**Figure 6:**
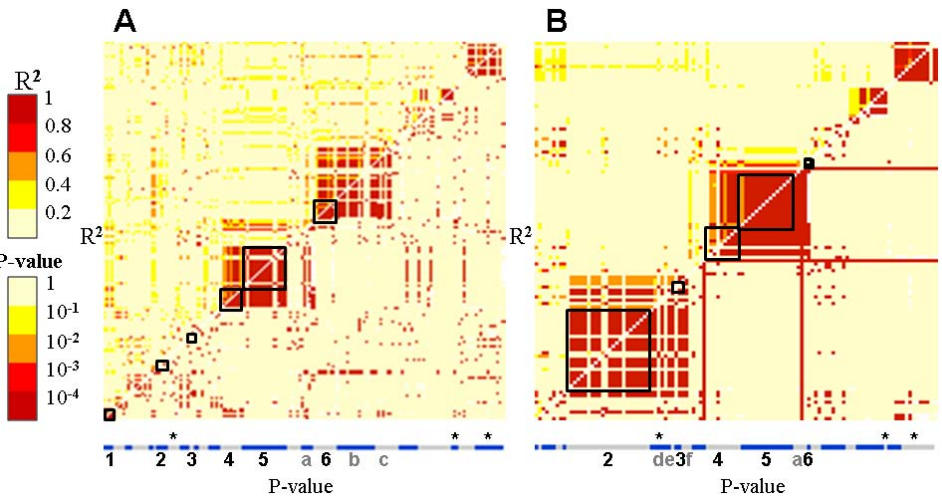
**Linkage disequilibrium along the second *flb *sub-region in the cultivated and wild compartments**. LD plots on R2 values (above the diagonal) and associated P-value (below the diagonal) along the second sub-region containing the 4 gene fragments under selection in the cultivated (**A**) and wild (**B**) compartments. The gene fragments re-sequenced are represented by alternate grey and blue boxes, which size is proportional to the number of polymorphic SNP used in the LD estimation. The gene fragments under selection are in black boxes and numbered as follows: "1" for VV05791A, "2" for VVC2897A, "3" for VV05785A, "4" for VVC2901A, "5" for VVC2885A and "6" for VVC2892A. The gene fragments in LD with these genes are pointed with small letter "a" for VV05782A, "b" for VV05781A, "c" for VV05780A, "d" for VV05786A, "e" for VVC2907A and "f" for VVC2891A. Gene fragments with SNP significantly associated with berry weight variation are highlighted with a star.

In order to check if there was linkage disequilibrium (LD) between the genes possibly under selection, LD was evaluated along the entire *flb *region. Two sub-regions were highlighted [additional file 7]. The first one, close to the telomere, contained two out of the eight genes possibly under selection (VVC2981A and VVC2946A), showed lower nucleotide diversity (Figure 5) and several gene fragments with no SNP in the wild pool. Moreover, in this sub-region, few significant LD was observed between the different gene fragments in both cultivated and wild pools [additional file 7]. The second sub-region contained six out of eight genes possibly under selection and showed high nucleotide diversity and a significant LD between and within some gene fragments in the cultivated and wild pools (Figure 5 and 6). Most of the SNPs found in the four out of the six genes possibly under selection showed intragenic LD, in the cultivated pool, and for two of them (VVC2901A and VVC2885A), an intergenic LD was found and extended with the adjacent gene fragment VV05782A (Figure 6). In the wild pool, only five out of the six gene fragments possibly under selection were polymorphic and could be used for the estimation of the LD in the second sub-region. Three of them showed intragenic and (excepted for VVC2897A) intergenic LD, together and with the gene fragment VV05782A as for the cultivated pool. Finally, the only gene possibly under selection showing significant nucleotide diversity difference between the two pools (VVC2897A) showed strong intragenic LD and with four adjacent gene fragments (VV05786A, VVC2907A, VV05785A and VVC2903A).

**Figure 7:**
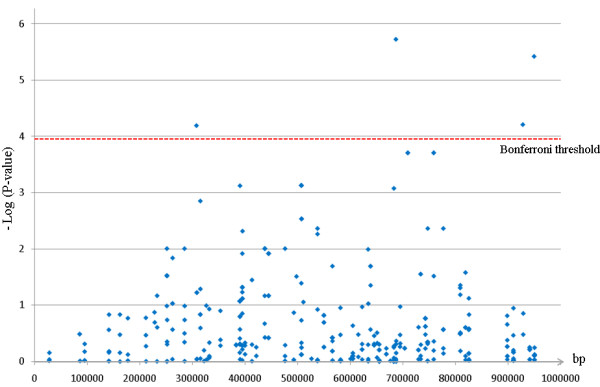
**Association tests for berry weight**. Level of association between SNP markers and the average berry weight along the *flb *region, in the cultivated *V. vinifera *sample. The Bonferroni threshold is equal to 1.12E-4.

We searched for associations in the set of cultivated genotypes between the average weight of mature berries and the 447 out of 554 SNPs from the *flb *region with an allelic frequency >0.05. Such significant associations (Figure 7; [additional file 8]) were detected for four SNPs in four gene fragments listed in the Table 5. None of them corresponded to the genes showing a significant deviation from neutrality of the Tajima's D parameter. However, a significant association was found with a non synonymous SNP from a gene fragment (VV05786A; Table 5) showing LD with the only gene fragment possibly under selection with a high nucleotide diversity in the wild pool than in the cultivated pool, VVC2897A.

**Table 5:** SNPs significantly associated with the variation of berry weight in the cultivated and wild pool of *Vitis vinifera*.

Fragment	Position of the SNP	P-value^$^	Feature	SNP type	SNP frequency	Putative function
VVC2966A	307,938	6.44E-05	CDS	[A/T]	5.00%	Unknown
VV05786A	687,166	1.87E-06	CDS	[A/G]*	9.00%	Protein kinase
VV05777A	928,516	6.18E-05	Intron	[T/G]	8.00%	Catalase
VV05775A	949,972	3.78E-06	Intron	[A/T]	9.00%	Ribosome biogenesis protein

**^$ ^**Significant P-value after Bonferroni correction (for α = 0.05)

* Non-synonymous mutation

## Discussion

With an initial objective to develop markers tightly flanking the *flb *mutation, 62 genomic regions were scanned for polymorphism along a 1.4 Mb region at the top of chromosome 18, where the mutation was previously located [21]. These regions were either genotyped or sequenced in the genotype carrying the mutation, Ugni Blanc mutant, its wild type (Ugni Blanc) and Chardonnay, which was the other parent of a full sib family segregating for the mutation. The sequenced fragments or markers were completely homozygous in Ugni Blanc and Ugni Blanc mutant, with one marker analyzed each 23 kb in average. Indeed, while analyzing the genome sequence of the heterozygous grapevine cultivar Pinot Noir, Velasco et al [43] showed that, like in other heterozygous species, the frequency of SNPs or INDELs varied along the grapevine genome and found some evidence for scarce quasi-homozygous areas. Here we describe a region of 1 Mb probably completely homozygous that raised two questions. First, as Velasco et al [43] showed that over 65 Mb of sequence are hemizygous in Pinot Noir, we wanted to check if our observations were due to a real homozygosity or to a deletion of a large portion of the top of chromosome 18 in one haplotype of Ugni Blanc. We addressed this issue by two different experiments (Figure 2), a qPCR estimation of the number of copies of a single gene in the homozygous area (*FL*) compared to genes elsewhere in the genome (three *HMGCoA *genes). The same number of copies was estimated for *FL *gene for Ugni Blanc mutant and Chardonnay which is heterozygous in this region. Second, a BAC-FISH hybridization on Ugni Blanc mutant metaphase chromosomes using a BAC clone located in the area was carried out and showed a signal on both homologous chromosomes. We therefore un-ambiguously demonstrated that our observations corresponded to a real homozygosity in Ugni Blanc mutant. This would be consistent with the fact that hemizygous regions identified by Velasco et al [43] would mainly correspond to stretches of repeated sequences, which is not the case of the *flb *region. These results raised the question whether this high level of homozygosity in Ugni Blanc mutant was restricted to the top of chromosome 18. The scoring of 480 SNPs [44] and 20 SSR ([45], V. Laucou personal communication) regularly spread along the genome showed that whereas this cultivar seems slightly more homozygous in average than for instance Cabernet Sauvignon, Syrah or Chardonnay, the near complete homozygosity observed in the *flb *region in Ugni Blanc mutant is not the rule on the rest of the genome and may be restricted to this region only. A mechanism which could explain the formation of such large homozygous region in a highly heterozygous out-crosser like grapevine would involve the repair of a DNA double-strand break [46]. When analyzing diversity in the cultivated germplasm, we observed that the cultivar Orbois was also completely homozygous for all fragments re-sequenced at the top of chromosome 18, and confirmed by qPCR assay that it was also due to real homozygosity (data not shown). Whatever its origin, this unexpected result made impossible the fine mapping of the mutation in the available segregating F1 population, which would necessitate the development of a F2 population.

Before having such a population available, we tested another possibility for reducing the interval carrying the *flb *gene, based on the fact that *flb *could be a gene selected during grape domestication. Indeed, the berry and seed phenotypes of Ugni Blanc mutant look like the phenotypes of wild *V. vinifera *seeds and berries [20,22]. We searched for signatures of selection in the *flb *region in a set of cultivated genotypes. For this purpose, we sequenced in 33 individuals (26 cultivated and 7 wild genotypes) (i) 69 gene fragments for a total of 34,355 kb along 948 kb in the *flb *region and (ii) 77 additional, totaling 48,874 kb spread along the 19 grapevine chromosomes. As already observed by Vezzulli et al [41], the nucleotide diversity was lower in average in the set of wild genotypes (π = 0.27) than in the set of cultivated genotypes (π = 0.35). This difference was increased in the *flb *region probably by the fact that a quarter of the fragments showed no sequence polymorphism at all among the wild genotypes whereas only one was in this case in the domesticated compartment. In the present work (but not in Vezzulli et al [42]) the sample of domesticated genotypes was selected after a comprehensive analysis of the world-wide largest collection of domesticated grapevine accessions [23] with the aim to retain a maximum of diversity. Unlike in Vezzulli et al [42], very few specific SNPs were found in the set of wild genotypes (six SNPs out of 554). All these observations, opposite to what has been observed in many other species [47,48], could be due to the fact that only seven to ten wild genotypes were sequenced in both studies and that their choice could not be driven by a maximization of the diversity along their complete natural area of growth. However, several surveys including accessions from the wild *V. vinifera *germplasm also showed this overall lower genetic diversity compared to the cultivated germplasm [49-51]. Indeed, small population sizes [52,53] as well as dioecy [54] could explain the observed reduced diversity in the wild *V. vinifera *gene pool, while multiple domestication events [50] and a continuous breeding process the larger diversity in the cultivated gene pool. All these results including ours will have to be confirmed with a larger sample of wild *V. vinifera*, taking into account all the geographic area where it grows and its overall genetic diversity.

Putative signatures of selection (Tajima's D parameter significantly deviating from neutrality associated with differences between the cultivated and wild pools in sequence diversity) in the cultivated pool of genotypes and SNPs showing a significant association with berry weight variation were found in the *flb *region (Figure 5). Moreover, LD was found in this region, the genes under selection presenting intragenic LD and intergenic LD with nearby genes (Figure 6), which strengthened the hypothesis that they might be under selection [48]. Like in Fournier-Level et al [55], no LD was however observed between the six out of eight genes possibly under selection. It is well known that the LD extent varies between the organisms and genomic regions [56]. In grapevine, a recent preliminary genome wide study confirmed the low extent of LD in average (less than 3-10 kb), suggesting a large effective size in the grapevine population at the origin of the current domesticated pool [57]. However, in recent history, vegetative propagation and long intervals between generations may have reduced the impact of recombination, maintaining extensive linkage disequilibrium in some regions under selection [58].

The significant genetic associations found between berry weight variation and SNPs in this region were not found in the fragments putatively under selection. It is still possible that one of these genes is involved into the berry weight variation and that the causative sequence polymorphism was not in the exon fragments sequenced. Indeed, trait variation is often due to sequence variation in regulatory regions (see for instance [9,11,55]) and such variant sites may be more tightly linked to a neighbor gene. Interestingly, the orthologs in Aradidopsis for the six out of eight gene with signature of selection (At1g76540 for VVC2946A, At1g76400 for VV05791A, At1g42970 for VVC2897A, At1g42540 for VV05785A, At5g43820 for VVC2901 and At1g20696 for VVC2892A) and for the two genes with SNPs associated with berry weight variation (At5g24306 for VV05786A and At1g42440 for VV05775A), are all expressed in flowers and showed a peak of expression at the beginning or during the flowering (http://urgv.evry.inra.fr/projects/FLAGdb++/HTML/index.shtml). In tomato, the genes involved into fruit size variation have been shown to be expressed in very early stages of its development, starting at floral development [2,11]. Finally, only one gene fragment, VVC2897A, might be under purifying selection in the cultivated pool with a major haplotype in cultivated pool and a LD extended to a neighbor gene which present a SNP significantly associated with berry weight variation (Figure 5 Figure 6). This would fit with the hypothesis that, like in tomato, the selection of a new haplotype by humans would have ensured the transition from berries with little flesh in wild grapevines to berries with more flesh in cultivated grapevines [2,11]. The real involvement of this gene into berry size variation and in the domestication syndrome remains however to be proven.

## Conclusions

While searching for SNP markers in coupling with the fleshless berry mutation, we observed the occurrence of a 1 Mb homozygous region, not associated with repetitive sequences, in the grapevine otherwise highly heterozygous genome. We demonstrated the feasibility to use BAC-FISH on the very small grapevine chromosomes and provided a specific probe for the identification of chromosome 18 on cytogenetic map. Using this method, we showed that the observed homozygosity was not due to a large deletion.

We then searched for signatures of domestication for berry weight along the *flb *region by re-sequencing 69 gene fragments in 26 domesticated and seven wild *V. vinifera *genotypes. We found putative signatures of selection associated with significant differences in nucleotide diversity between the cultivated and the wild pool only in one gene (VVC2897A) and also SNPs significantly associated with berry weight variation in three other genes among which one is in DL with VVC2897A. The involvement of these four genes into berry weight variation in grapevine remains to be proved by further functional experiments. In addition, we detected 554 SNPs along the *flb *region. These polymorphisms could serve to develop a genotyping chip useful for a future fine mapping of the *flb *gene in a F2 population and for the analysis of genetic diversity in larger sets of wild and cultivated genotypes.

## Authors' contributions

CH designed the primers for fragment re-sequencing, participated to their sequencing, analyzed the results, chose the BAC and prepared the root tips for the FISH, draft and corrected the paper. RB, M-CLP and DB were responsible for the sequencing of the fragments. RB and AC participated to the sequence analysis and made the RT-PCR experiment. JC and LT analyzed the SSR and genotyped SNP polymorphism. CG and AD helped with bio-informatics (scripts, database queries...). J-PP and RB generated the set of reference fragment sequences along the genome. PT, TL, and AND provided the DNA for the core-collection and plant phenotypes. M-SV and OC did the BAC-FISH experiments. A-FAB design the experiment, supervised it, drafted and corrected the manuscript. All authors read and approved the final manuscript.

## Supplementary Material

Additional file 1 **supplemental table S1. Plant material**. List of the grapevine accessions used in the study, with their average berry weight at maturity. Click here for file

Additional file 2 **supplemental table S2. Markers and sequence fragments along the *flb *region**. Description: List and localization on the grapevine genome sequence of the gene fragments sequenced (SEQ) and of the markers used for genotyping (SSR, CAPS, SNP) together with the primers used for amplification. Click here for file

Additional file 3 **supplemental table S3. Sequence fragments randomly spread along the genome**. List and localization on the grapevine genome sequence of the random gene fragments sequenced spread all over the genome, diversity and Tajima's D parameters obtained in the cultivated and the wild pools of *Vitis vinifera*. Click here for file

Additional file 4 **supplemental table S4. qPCR validation of homozygosity in the *flb *region in Ugni Blanc mutant**. Estimation of the initial number of DNA quantity of the *FL *gene and of the *HMGCoA *gene family in Pinot Noir (PN777), Chardonnay (CHA), Ugni Blanc mutant (UBM) and Ugni Blanc (UB), before and after normalization by the result obtained for the *HMGCoA *genes. Click here for file

Additional file 5 **supplemental table S5. Sequence diversity along the *flb *region in cultivated and wild grapevines**. Parameter of diversity obtained for the 69 genome fragments at the top of chromosome 18 in the cultivated and the wild compartment of *Vitis vinifera *and details of the shared and specific insertions/deletions (INDELs) and segregating SNP sites in wild *V. vinifera *genotypes and table and wine cultivars of domesticated *V. vinifera*, numbers of shared and unshared SNPs or INDELs between wild and cultivated genotypes are also indicated. Click here for file

Additional file 6 **supplemental figure S1. Nucleotide diversity in the cultivated (table and wine) and wild compartments along the *flb *region**. Nucleotide diversity (π) in the table grapes (green line), the wine grapes (purple line) and the wild grapes (blue line) along the *flb *region. Click here for file

Additional file 7 **supplemental figure S2. Linkage disequilibrium along the *flb *region in cultivated and wild compartments**. LD plots on R^2 ^values (above the diagonal) and associated P-value (below the diagonal) along the entire *flb *region in cultivated (A) and wild compartments (B). The gene fragments re-sequenced are represented by alternate grey and blue boxes, which size is proportional to the number of polymorphic SNP used in the LD estimation. The black arrow represents the orientation of the region from the telomere (on the left) to the centromere. Click here for file

Additional file 8 **supplemental table S6. List of gene fragments and their associated number of SNPs used for the estimation of LD in the *flb *region, in cultivated and wild *V. vinifera *pools**. Click here for file

## Aknowledgements

This study was supported by INRA, ANR and the French Ministry of Research. We thank Isabelle Le Clainche, Audrey Weber, Sylvain Santoni, Christophe Lepage and all the team of the Vassal germplasm collection for technical assistance; Stéphane Nicolas, Aurélie Siberchicot, Christine Cierco and Brigitte Mangin for providing a R routine for LD calculation and helpful discussions; Marie-Laure Martin Magniette for help in the statistical analysis, Dr Mark R. Thomas from the CSIRO Plant Industry, Australia for providing unpublished DNA marker information and Loïc Le Cunff for helpful discussions.
