# Supplementary material for: Patterns of sequence polymorphism in the fleshless berry locus in cultivated and wild Vitis vinifera accessions
Source: BMC Plant Biol. 2010 Dec 22;10:284. doi: 10.1186/1471-2229-10-284 (PMC3022909; doi:10.1186/1471-2229-10-284)
Supplement: Additional file 1 — supplemental table S1. Plant material. List of the grapevine accessions used in the study, with their average berry weight at maturity. [file 1471-2229-10-284-S1.PDF]

Table S1. Plant material.

Name	Accession number	Wine (W)/Table (T) /wild (S)	Average berry weight (g)$^{\$}$
Ak ouzioum tagapskii*	2897Mtp1	T	3.8
Araklinos*	1805Mtp1	W	2.1
Assyl kara*	2505Mtp1	T	3.0
Cabernet franc*	324Mtp1	W	1.7
César*	225Mtp2	W	2.3
Chirai obak*	1186Mtp1	T	3.6
Chouchillon*	192Mtp1	W	1.8
Espadeiro tinto*	1498Mtp1	W	1.9
INRA Colmar Lignée PN40024	0Mtp1840	_	_
Kapistroni tétri hermaphrodite (Coll. Kichinev)*	0Mtp567	T	3.5
Lameiro*	0Mtp636	W	1.1
Katta-kourgan*	556Mtp1	T	9.2
Plant du Maroc E (Collection Meknès)*	2158Mtp1	T	5.9
Médouar*	0Mtp715	T	5.1
Mehdik*	2082Mtp1	T	2.6
Muscat à petits grains blancs*	555Mtp2	W	2.3
Orbois*	294Mtp1	W	1.7
Orlovi nokti*	2461Mtp1	T	4.3
Pervenetz praskoveïsky*	2651Mtp2	T	2.1
Kichmich tcherni*	0Mtp583	T	2.5
Pletchistik*	2652Mtp1	W	2.0
Sultanine*	1566Mtp2	T	1.9
Syrah*	150Mtp3	W	1.5
Tsitsa Kaprei*	2471Mtp1	T	4.1
Tsolikouri*	0Mtp1150	W	2.4
Variété d'oasis Bou Chemma 46*	0mtp1557	T	?
Voskeat*	2511Mtp1	W	?
Lambrusque Campmarcel 2	8500Mtp23	S	1.0
Lambrusque Colmar femelle	8500Mtp9	S	1.0
Lambrusque Dirmstein mâle	8500Mtp38	S	_
Lambrusque Grésigne 1	8500Mtp124	S	1.0
Lambrusque Pic Saint-Loup 2	8500Mtp113	S	1.0
Lambrusque Sejnene 1	8500Mtp31	S	1.0
Lambrusque Teulere	0Mtp1323	S	1.0
Chardonnay	199Mtp12	W	1.7
Pinot Noir clone ENTAV-INRA 777	ENTAV1617	W	1.4
Ugni Blanc	74mtp31	W	2.4
Ugni Blanc mutant (fleshless berry)	0Mtp1596	_	0.3

$^{\$}$ The average berry weights at maturity were retrieved from unpublished experiments of C. Houel, L. Torregrosa and from the data archives of the Domain of Vassal

* genotypes maximizing the genetic diversity of the cultivated *Vitis vinifera* compartment, from Lecunff et al [23]
