# Supplementary material for: Patterns of sequence polymorphism in the fleshless berry locus in cultivated and wild Vitis vinifera accessions
Source: BMC Plant Biol. 2010 Dec 22;10:284. doi: 10.1186/1471-2229-10-284 (PMC3022909; doi:10.1186/1471-2229-10-284)
Supplement: Additional file 4 — supplemental table S4. qPCR validation of homozygosity in the flb region in Ugni Blanc mutant. Estimation of the initial number of DNA quantity of the FL gene and of the HMGCoA gene family in Pinot Noir (PN777), Chardonnay (CHA), Ugni Blanc mutant (UBM) and Ugni Blanc (UB), before and after normalization by the result obtained for the HMGCoA genes. [file 1471-2229-10-284-S4.PDF]

Table S4. qPCR validation of homozygosity in the flb region in Ugni Blanc mutant. Estimation of the initial number of DNA quantity of the FL gene and of the HMGCoA gene family in Pinot Noir (PN777), Chardonnay (CHA), Ugni Blanc mutant (UBM) and Ugni Blanc (UB), before and after normalization by the result obtained for the *HMGCoA* genes.

	FL		***HMGCoA***		***FL* normalized**	
	Qty Mean	Qty StdDev	Qty Mean	Qty StdDev	Qty Mean	Qty StdDev
**PN777**	1.54	0.15	1.37	0.06	**1.13**	**0.17**
**CHA**	2.43	0.09	2.28	0.07	**1.07**	**0.08**
**UBM**	2.59	0.14	2.38	0.05	**1.09**	**0.08**
**UB**	1.79	0.03	1.47	0.05	**1.23**	**0.06**
