# Supplementary figures and images for: Patterns of sequence polymorphism in the fleshless berry locus in cultivated and wild Vitis vinifera accessions
Source: BMC Plant Biol. 2010 Dec 22;10:284. doi: 10.1186/1471-2229-10-284 (PMC3022909; doi:10.1186/1471-2229-10-284)

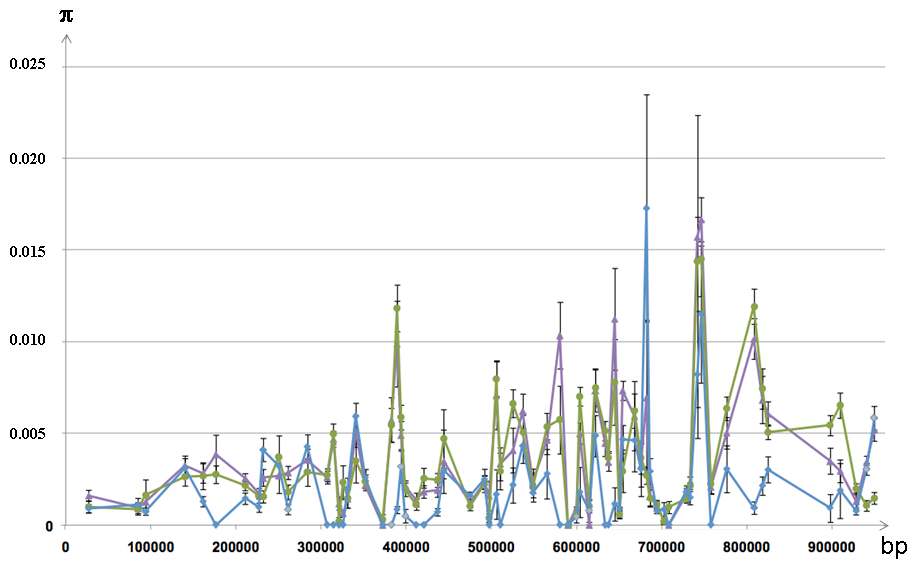

Supplement: Additional file 6 — supplemental figure S1. Nucleotide diversity in the cultivated (table and wine) and wild compartments along the flb region. Nucleotide diversity (π) in the table grapes (green line), the wine grapes (purple line) and the wild grapes (blue line) along the flb region. [file 1471-2229-10-284-S6.TIFF]

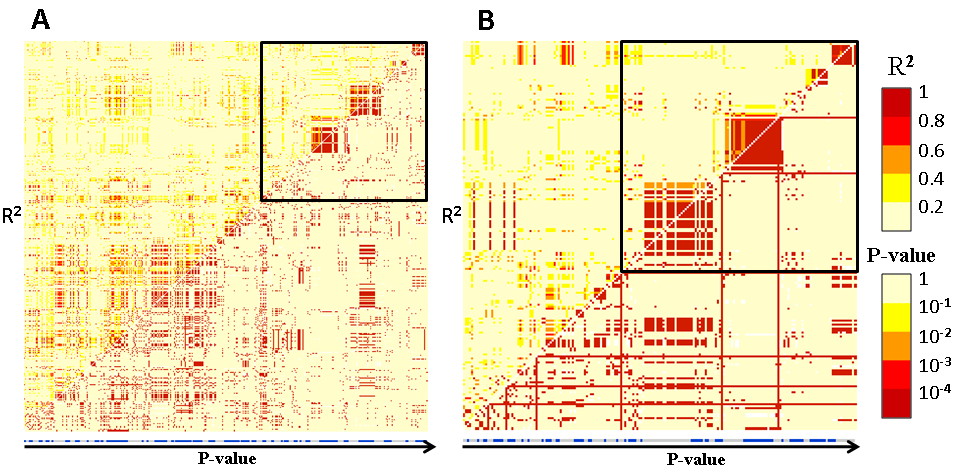

Supplement: Additional file 7 — supplemental figure S2. Linkage disequilibrium along the flb region in cultivated and wild compartments. LD plots on R2 values (above the diagonal) and associated P-value (below the diagonal) along the entire flb region in cultivated (A) and wild compartments (B). The gene fragments re-sequenced are represented by alternate grey and blue boxes, which size is proportional to the number of polymorphic SNP used in the LD estimation. The black arrow represents the orientation of the region from the telomere (on the left) to the centromere. [file 1471-2229-10-284-S7.TIFF]
