# Supplementary material for: Patterns of sequence polymorphism in the fleshless berry locus in cultivated and wild Vitis vinifera accessions
Source: BMC Plant Biol. 2010 Dec 22;10:284. doi: 10.1186/1471-2229-10-284 (PMC3022909; doi:10.1186/1471-2229-10-284)
Supplement: Additional file 8 — supplemental table S6. List of gene fragments and their associated number of SNPs used for the estimation of LD in the flb region, in cultivated and wild V. vinifera pools. [file 1471-2229-10-284-S8.PDF]

**Table S6.** List of gene fragments and their associated number of SNPs used for the estimation of LD in the *flb* region, in cultivated and wild *V. vinifera* pools.

| | | Number of SNP marker used for LD estimation | |
|---|---|---|---|
| **gene name (12X)** | **primer nane** | **cultivated pool** | **wild pool** |
| GSVIVT00013479001 | VVC2985A | 3 | 1 |
| GSVIVT00013476001 | VVC2982A | 3 | 2 |
| GSVIVT00013475001[$] | VVC2981A | 4 | 1 |
| GSVIVT00013472001 | VV05807A | 6 | 4 |
| GSVIVT00013471001 | VV05806A | 4 | 1 |
| GSVIVG00013469001 | VVC2975A | 3 | _ |
| GSVIVT00013468001 | VVC2974A | 4 | 2 |
| GSVIVT00013467001 | VVC2973A | 2 | 2 |
| GSVIVT00013466001 | VVC2972A | 2 | 3 |
| GSVIVT00013462001 | VVC2970A | 12 | 5 |
| GSVIVT00013461001 | VV05805A | 5 | 2 |
| GSVIVT00013460001 | VV05804A | 5 | 4 |
| GSVIVT00013458001 | VVC2966A* | 5 | _ |
| GSVIVT00013456001 | VVC2965A | 7 | _ |
| GSVIVT00013455001 | VV05803A | 2 | _ |
| GSVIVT00013454001 | VVC2963A | 6 | _ |
| GSVIVT00013453001 | VVC2962A | 5 | 3 |
| _ | VV05802A | 1 | 1 |
| GSVIVT00013452001 | VV05801A | 3 | 2 |
| GSVIVT00013450001 | VVC2956A | 3 | _ |
| GSVIVT00013449001 | VVC2955A | 22 | 1 |
| GSVIVT00013448001 | VVC2954A | 14 | 6 |
| GSVIVT00013446001 | VVC2953A | 2 | 1 |
| GSVIVT00013445001 | VVC2951A | 3 | _ |
| GSVIVT00013443001 | VVC2950A | 2 | _ |
| _ | VVC2947A | 6 | 1 |
| GSVIVT00013440001[$] | VVC2946A | 13 | 3 |
| GSVIVT00013438001 | VVC2944A | 4 | 2 |
| GSVIVT00013436001 | VVC2943A | 3 | 2 |
| GSVIVT00013435001 | VVC2942A | 1 | _ |
| GSVIVT00013434001 | VVC2940A | 17 | 7 |
| GSVIVT00013433001 | VV05798A | 1 | _ |
| GSVIVT00013430001 | VVC2935A | 10 | 5 |
| GSVIVT00013427001 | VVC2933A | 6 | 2 |
| GSVIVT00013425001 | VV05796A | 7 | 2 |
| GSVIVT00013423001 | VVC2929A | 9 | 6 |
| GSVIVT00013421001 | VVC2927A | 16 | _ |
| GSVIVT00013417001 | VVC2921A | 1 | 1 |
| GSVIVT00013416001 | VVC2920A | 9 | 5 |
| GSVIVT00013414001 | VV05793A | 2 | 1 |
| GSVIVT00013413001 | VVC2918A | 5 | 3 |
| GSVIVG00013411001 | VVC2916A | 6 | _ |

| | | | |
|---|---|---|---|
| GSVIVT00013410001$ | VV05791A | 4 | _ |
| GSVIVT00013409001 | VVC2914A | 5 | 1 |
| GSVIVT00013408001 | VV05790A | 3 | 1 |
| GSVIVT00013407001 | VVC2912A | 7 | 4 |
| GSVIVG00013406001 | VV05788A | 2 | 2 |
| GSVIVT00013404001 | VVC2910A | 1 | 1 |
| GSVIVT00013403001$ | VVC2897A | 5 | 24 |
| GSVIVT00013402001 | VV05786A* | 5 | 6 |
| GSVIVT00013401001 | VVC2907A | 4 | 1 |
| GSVIVT00013400001$ | VV05785A | 2 | 2 |
| GSVIVT00013399001 | VVC2905A | 2 | _ |
| GSVIVT00013397001 | VVC2891A | 4 | 3 |
| GSVIVT00013395001 | VV05783A | 5 | 4 |
| GSVIVT00013394001$ | VVC2901A | 9 | 10 |
| GSVIVT00013393001$ | VVC2885A | 19 | 15 |
| GSVIVT00013392001 | VVC2884A | 6 | _ |
| GSVIVT00013389001 | VV05782A | 5 | 4 |
| GSVIVT00013385001$ | VVC2892A | 10 | 1 |
| GSVIVT00013382001 | VV05781A | 16 | 3 |
| GSVIVT00013381001 | VV05780A | 10 | 7 |
| GSVIVT00013373001 | VV05779A | 8 | 3 |
| GSVIVT00013370001 | VV05778A | 14 | 8 |
| GSVIVT00013367001 | VV05777A* | 3 | 1 |
| GSVIVT00013365001 | VV05776A | 7 | 4 |
| GSVIVT00013363001 | VV05775A* | 11 | 9 |

$ genes showing a significant deviation from neutrality of Tajima's D parameters

* genes with SNPs significatively associated with berry weight variation
